# Vertical two-phase flow regimes in an annulus image dataset - Texas A&M university

**DOI:** 10.1016/j.dib.2024.111245

**Published:** 2025-01-04

**Authors:** Kaushik Manikonda, Chinemerem Obi, Aarya Abhay Brahmane, Mohammad Azizur Rahman, Abu Rashid Hasan

**Affiliations:** a Texas A&M University, College Station, TX 77840, United States; b Georgia Institute of Technology, Atlanta, GA 30332, United States; c Dell Technologies, Round Rock, TX 78664, United States; d Texas A&M University at Qatar, Education City, Al Rayyan, Qatar; e Hamad Bin Khalifa University, Education City - Gate 8, Ar-Rayyan, Qatar

**Keywords:** Gas-liquid Two-phase flow, Machine vision, Multi-phase flow, Machine learning, Computer vision

## Abstract

The Vertical Two-Phase Flow Regimes in an annulus Image Dataset, generated at Texas A&M University, presents an extensive collection of high-resolution images capturing various gas-liquid two-phase flow dynamics within a vertical flow setup. This dataset results from meticulous experimental work in the 140 ft Tower Lab, utilizing a combination of water and air flows to simulate real-world conditions and employing high-quality video recordings to document flow regime transitions. Designed to support research in fluid dynamics, machine vision, and computational modeling, the dataset offers valuable resources for developing machine vision models for accurate regime detection and differentiation, enhancing the fidelity of computational fluid dynamics simulations, and facilitating the estimation of critical flow parameters. Despite its comprehensive nature, the dataset notes limitations such as the absence of annular flow regime images and its exclusive focus on vertical flow conditions.

Specifications Table

**Table:** 

Subject	Petroleum Engineering
Specific subject area	Fluid Flow and Transfer Processes, Computer Vision, and Pattern Recognition
Type of data	Raw Images
Data collection	Our experimental data were generated using a specialized setup in the 140 ft Tower Lab, Richardson Petroleum Engineering Building, Texas A&M University. This facility houses a unique vertical flow loop system comprising a 140 ft tall, 5.5” inner diameter clear PVC pipe and a central 2-3/8” outer diameter drill pipe, creating an annular space for studying gas-liquid two-phase flow dynamics. Water and air were introduced into this system to mimic real-world flow conditions. A combination of a 200 GPM pump for water and a compressor for air, controlled through a sophisticated data acquisition and control system using NI-9188XT and LabVIEW software, allowed for precise manipulation and monitoring of the flow. High-resolution video capture of the flow regimes was achieved with cameras positioned along the tower, complemented by data from pressure transducers and Coriolis meters for continuous pressure and flow rate monitoring. This setup enabled a systematic investigation of flow dynamics, including bubble migration and flow regime transitions, under controlled laboratory conditions, forming the basis of our comprehensive dataset.
Data source location	Harold Vance Department of Petroleum Engineering, Texas A&M University Richardson Petroleum Engineering Building, 3116 TAMU, 245 Spence St, College Station, TX 77843 Country: United States of America GPS Coordinates: 30.61908391218633, -96.33905533862557
Data accessibility	Repository name: Vertical Two-Phase Flow Regimes in an Annulus Image Dataset - Texas A&M University Data identification number: 10.17632/nxncbzzz38.2 Direct URL to data: https://data.mendeley.com/datasets/nxncbzzz38/2

## 1 Value of the Data

• Gas-liquid two-phase flow is ubiquitous in many fields, such as petroleum engineering, nuclear engineering, medicine, and mechanical engineering, among many others. Recognizing a two-phase flow regime is crucial wherever it is present. As such, the proposed image dataset can aid in developing machine vision models that can accurately detect a two-phase flow regime when it occurs, especially in annular channels.
• This dataset provides a comprehensive collection of high-resolution images across different two-phase flow regimes (bubbly, slug, churn, Taylor Bubble) in a vertical flow setup. It enables researchers and engineers to study the intricate dynamics of gas-liquid flows, enhancing understanding and modeling of two-phase flow in an annulus phenomenon.
• Recognizing and differentiating various two-phase flow regimes like bubbly flow, slug flow, churn flow, or Taylor bubble flow is crucial whenever two-phase flow is involved. Hence, the proposed image dataset with clear flow regime divisions and tags can aid in developing machine vision models that can accurately distinguish various two-phase flow regimes.
• The dataset serves as a valuable benchmark for validating CFD simulations of two-phase flows. By comparing simulation results with real flow images, researchers can improve the fidelity of simulation tools, which are essential for designing efficient and safe flow systems without the need for extensive physical prototyping.
• In many gas-liquid two-phase flow scenarios, it is critical to accurately estimate flow parameters like liquid holdup, gas superficial velocity, liquid superficial velocity, and bubble lengths. Many images in the proposed dataset have measurement scales next to the bubbles, thus enabling researchers to use this dataset in training machine vision models that can reliably estimate these two-phase flow parameters.
• There are very few experimental data in the literature for two-phase flow through annuli. This data set, gathered in an annulus, is therefore, very valuable.

## 2 Background

This comprehensive dataset of gas-liquid two-phase flow regimes was motivated by the critical need to enhance understanding and modeling capabilities in scenarios where these flows are prevalent. This includes oil-well drilling and gas kick scenarios. The theoretical foundation for this dataset lies in the complex interplay between gas and liquid phases in vertical flow conditions, which significantly impacts the efficiency, safety, and environmental footprint of oil extraction processes. Specifically, we aimed to provide a detailed visual and empirical basis to support research into gas kick modeling, slug flow, bubble migration time estimations, two-phase flow parameter calculations, and gas kick expansion modeling, among others. By documenting various flow regimes such as Taylor bubble, bubbly, slug, and churn flows within a controlled environment, our dataset offers a unique value to ongoing research efforts. It complements existing theoretical [[1], [2], [3]] and experimental [[4], [5], [6], [7]] studies by providing a high-fidelity visual reference that can be used to verify models and simulations, thereby enhancing the accuracy of holdup calculations and other related parameters during two-phase flow regimes.

## 3 Data Description

The dataset encompasses a meticulously categorized collection of images capturing various two-phase flow regimes within a gas-liquid system. The dataset comprises 267 Taylor bubble images, 278 images of Bubbly flow, 285 of Slug flow, and 117 images documenting Churn flow, derived from experimental runs conducted under both static and dynamic conditions. These images are systematically organized into four folders: Taylor, Slug, Churn & Bubbly. The folder structure is as follows:

The naming convention of the images within these folders is meticulously designed to reflect the specific experimental conditions, including pressure (psig), flow rate (gpm for dynamic conditions), floor number, prefix of “CO2” for CO2 flow and observation time (seconds), facilitating easy identification and retrieval for analysis. This structured approach ensures a comprehensive and navigable dataset, invaluable for advancing research in two-phase flow dynamics. The image naming convention is as follow:

**Table:** 

Condition	Image Name Format
Air flow in static condition	psig_sec_floor_px; where x is the image number in that condition
Air flow in dynamic condition	psig_gpm_sec_floor_px; where x is the image number in that condition
CO2 flow in static condition	Co2_psig_sec_floor_px; where x is the image number in that condition
CO2 flow in dynamic condition	Co2_psig_gpm_sec_floor_px; where x is the image number in that condition

**Fig. 1:**
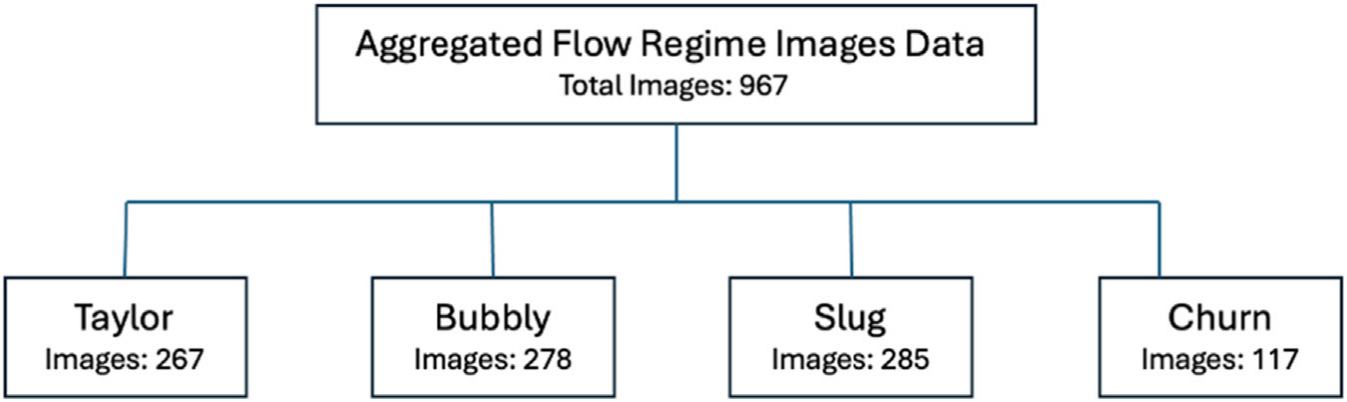
Folder structure diagram.

**Fig. 2:**
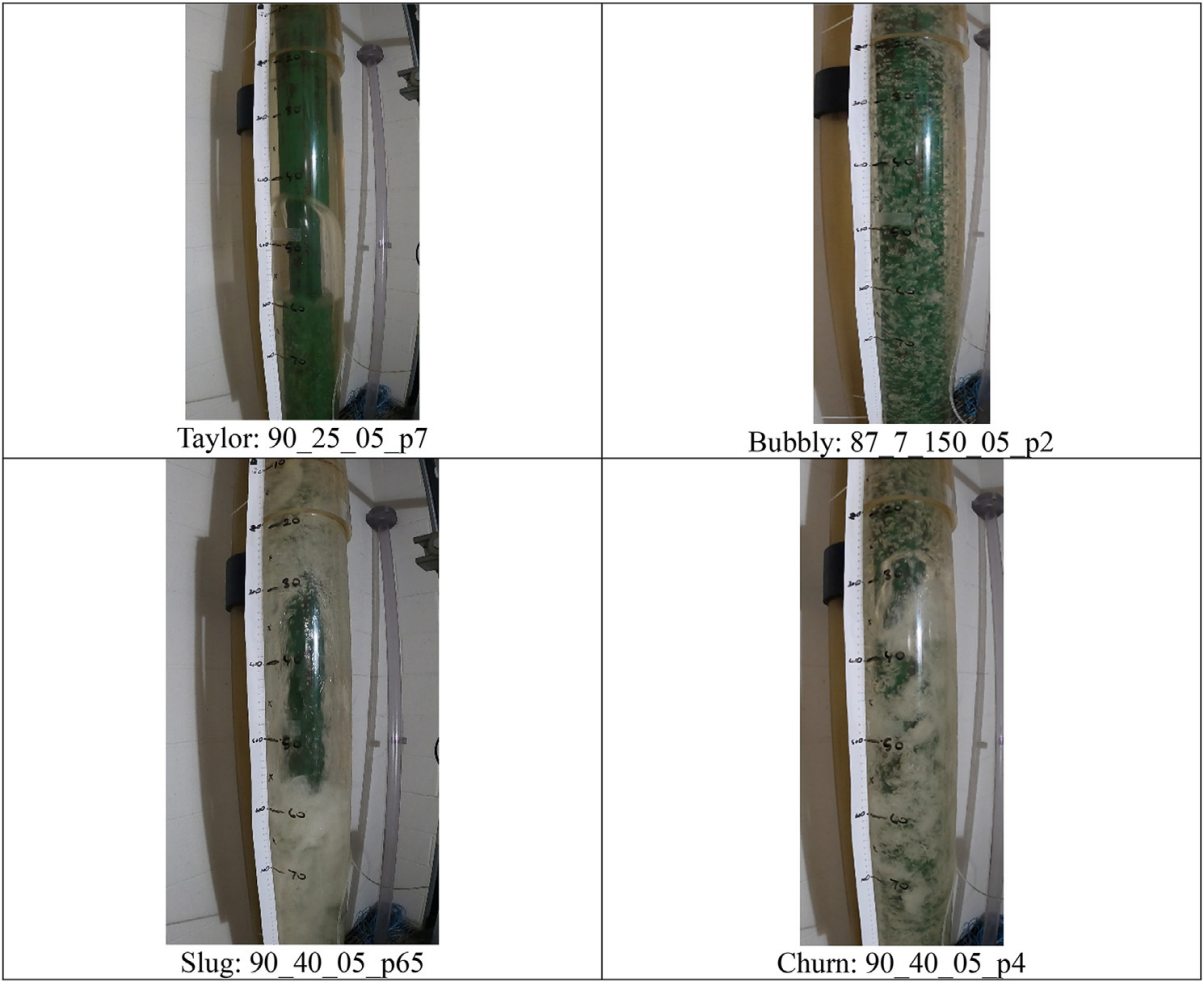
Example images from all Four flow regimes.

**Fig. 3:**
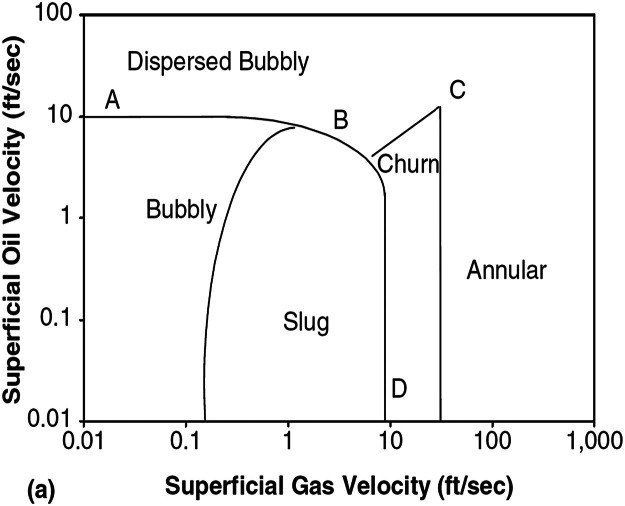
Vertical and inclined two-phase flow regime map presented by Hasan and Kabir [1]

Fig. 1, Fig. 2, below shows example of images across the four different (Taylor, Bubbly, Churn & Slug) two-phase flow regimes. The images in the example are selected at random. Image number is mentioned besides the type of flow regime:

For additional context, Fig. 3, below shows the well-accepted vertical two-phase flow regime map proposed by Hasan & Kabir [1].

**Fig. 4:**
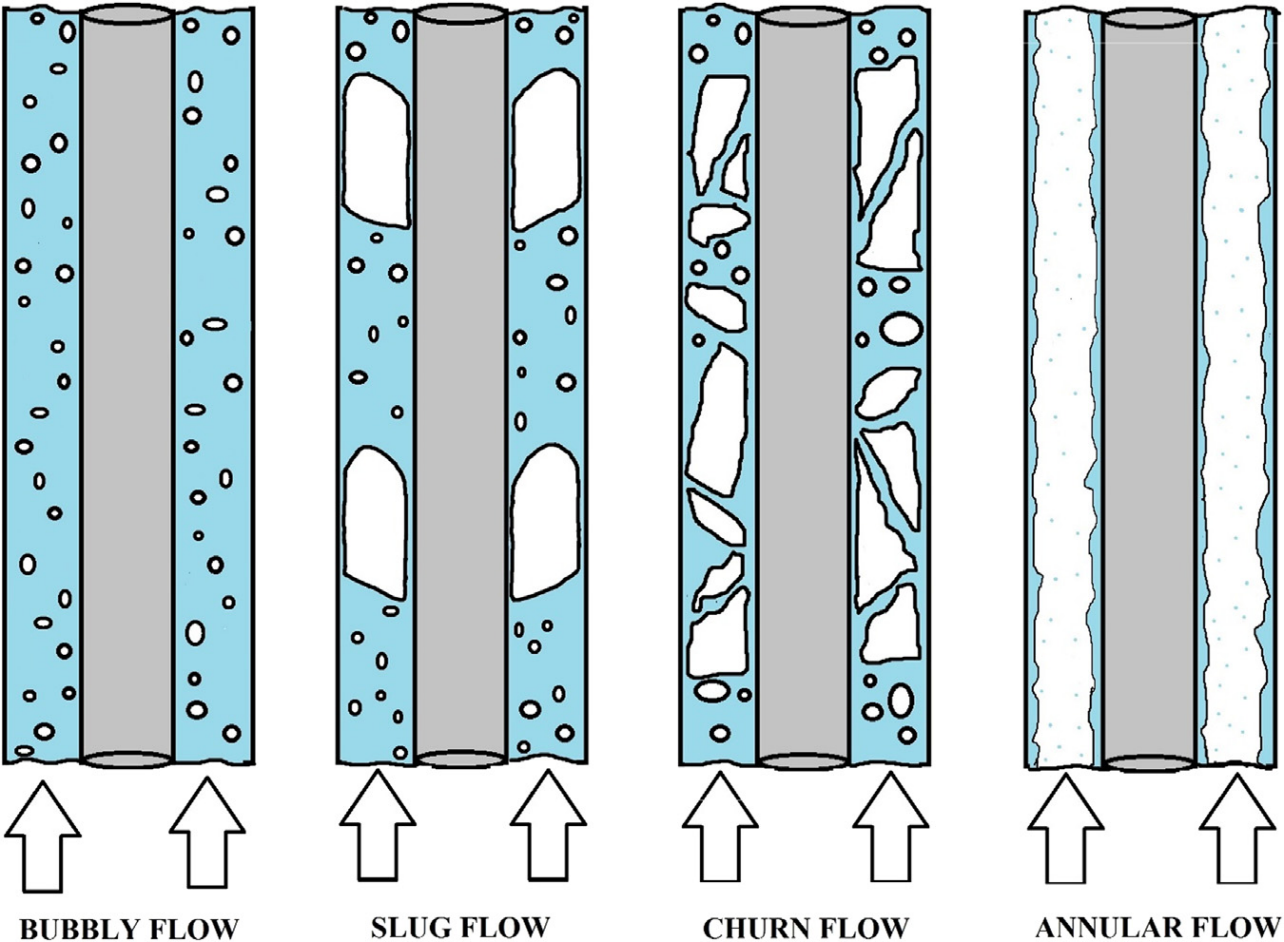
Bubbly, Slug, Churn, and annular flow regimes in vertical annular channels, depicted by Manikonda et al., [2]

Fig. 4, below diagrammatically depicts some of the different two-phase flow regimes we can expect in vertical, gas-liquid, flows through annular channels.

## 4 Experimental Design, Materials and Methods

**Fig. 5:**
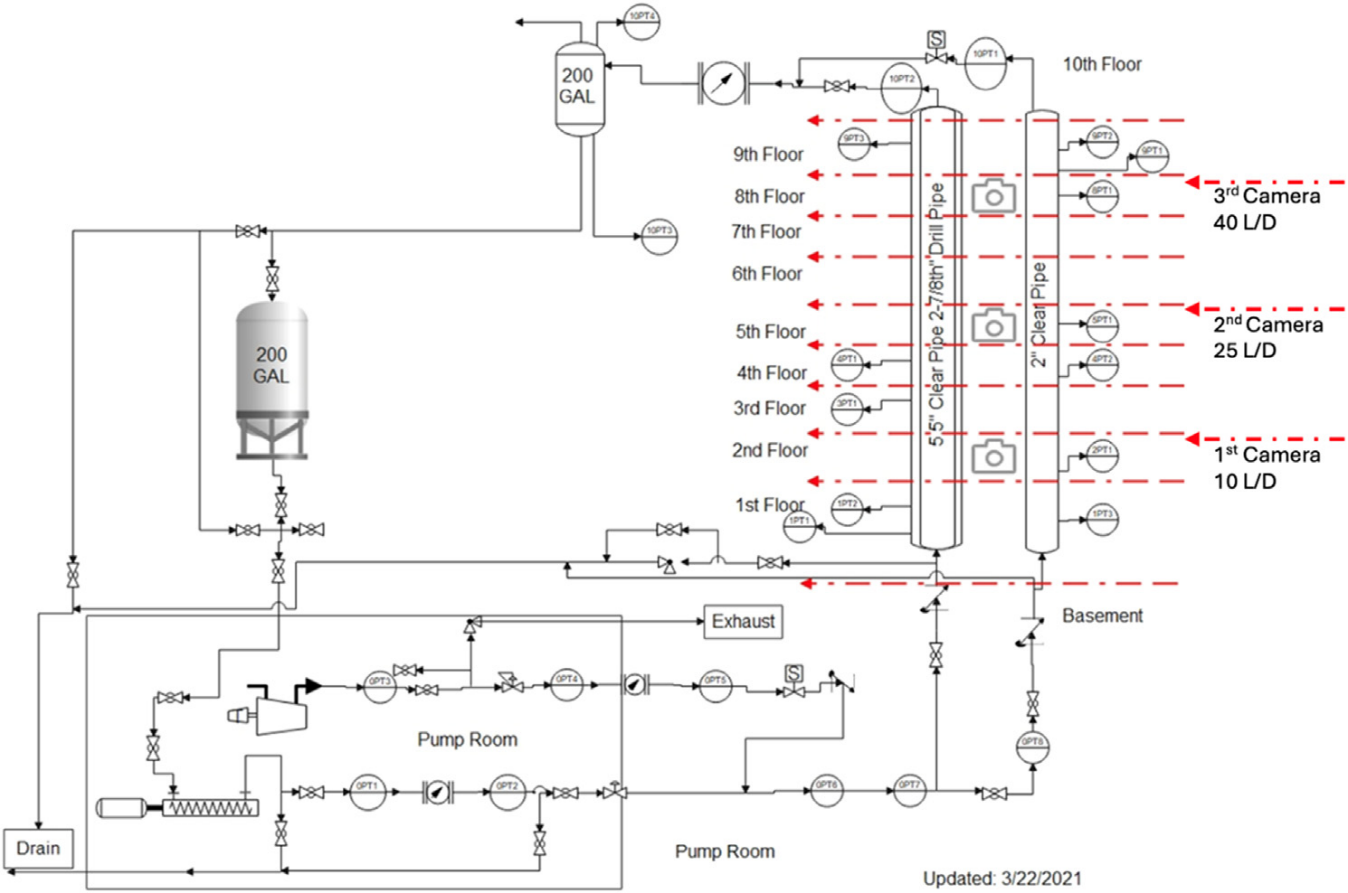
Tower lab schematic P&ID

We conducted our image requested"?> experiments at the 140-ft Tower Lab, located within the Richardson Petroleum Engineering Building at Texas A&M University. The lab's equipment features a 140-foot-tall flow loop consisting of a 5.5-inch inner diameter clear PVC pipe with a 2-3/8-inch outer diameter drill pipe running through its center to form an annular flow space. This configuration emulates the conditions found in actual oil wells, enabling us to explore the dynamics of gas-liquid two-phase (annulus) flow in a controlled laboratory setting. Fig. 5, below presents a detailed schematic of the Tower Lab.

**Fig. 6:**
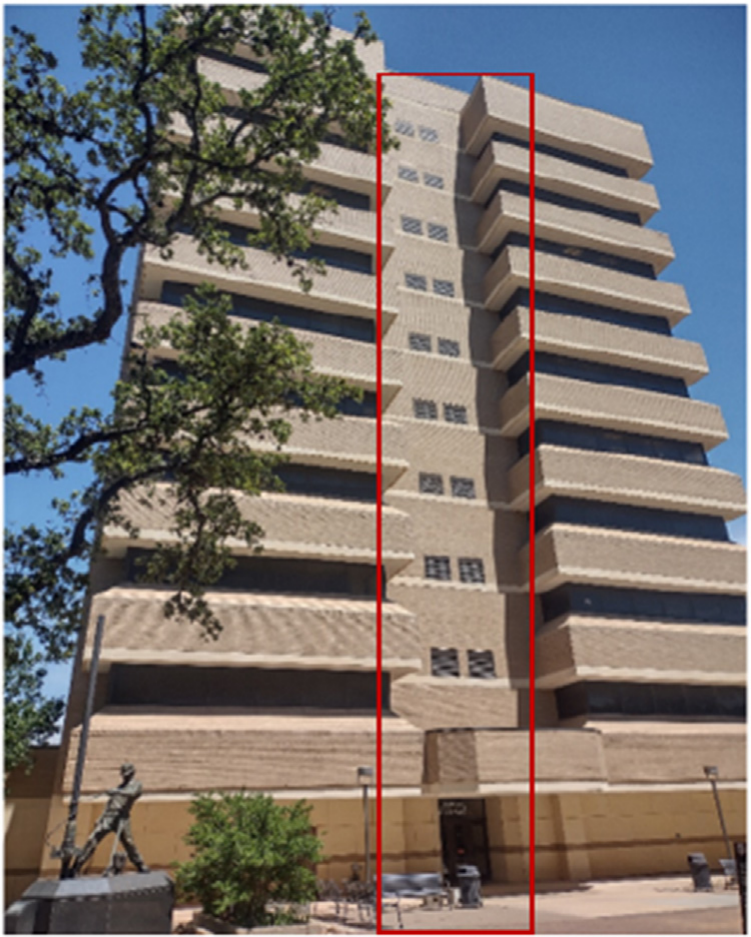
Richardson petroleum building (Tower lab section in red).

**Fig. 7:**
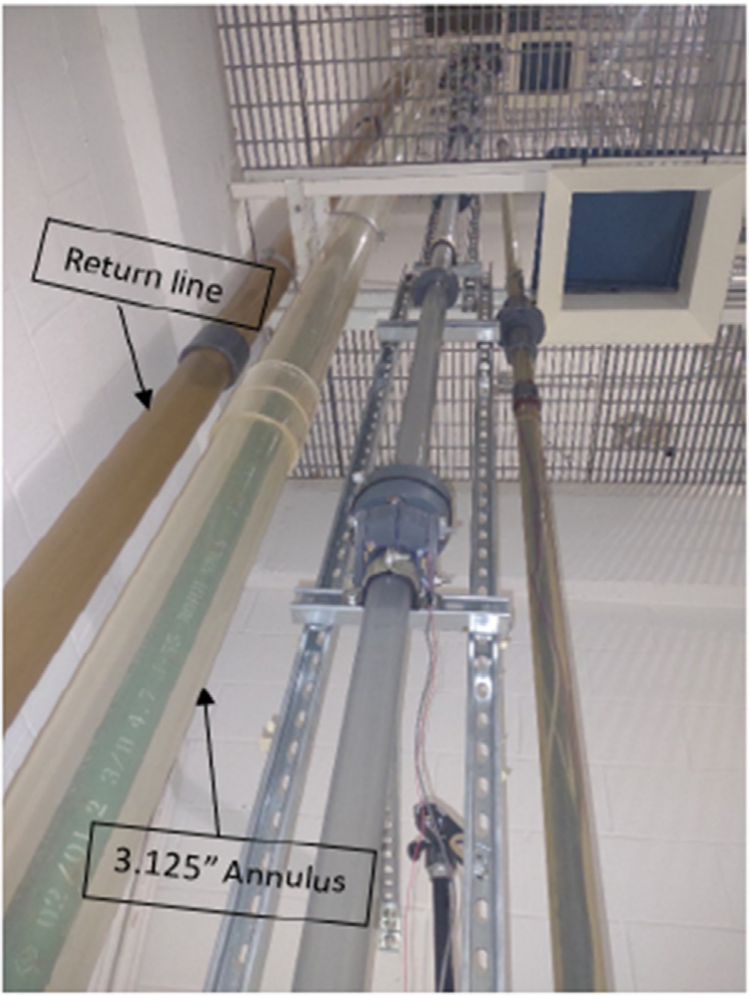
Inner (bottoms up) view of tower lab.

Figs. 6, and 7, below show the tower lab from the basement-up and its scale when compared to the Richardson Petroleum Engineering Building.

### 4.1 Materials and Methods

Flow Loop Configuration: We introduced water and air into the flow loop to mimic two-phase flow conditions. A 200 GPM pump, connected to a 2-inch steel galvanized pipe, supplied water from the bottom. We supplied air using a compressor through a 3/4-inch galvanized steel line, regulating the flow with a solenoid valve and monitoring it with pressure transducers and a mass flow meter.

**Data Acquisition and Control**: A NI-9188XT system, located in the basement and interfaced with LabVIEW software, managed the system's data acquisition and control. This arrangement allowed for precise adjustments in gas injection and water circulation within the flow loop, employing a solenoid valve for gas and a combination of a variable frequency drive (VFD) and an air-actuated control valve for water flow.

**Video Recording and Analysis**: During the experiments, high-quality videos of the gas-liquid two-phase flow within the annular space were captured using three cameras positioned at equal (length to diameter ratio) intervals along the tower (10 L/D, 25 L/D, 40 L/D). These recordings offered detailed observations of bubble migration and flow regime transitions, serving as the foundation of our dataset. The visual sections were also graduated with a measuring tape to ease estimation of travel distance and subsequently velocities traverse the Tower lab.

**Sensor Deployment**: We installed multiple pressure transducers throughout the flow loop and Coriolis meters (upstream and downstream) to continuously monitor pressure and flow rates, thus ensuring a thorough examination of the flow dynamics within the Tower lab.

**Experimental Procedure**: Our procedure entailed several steps, including filling the water tank, initiating water and air flow, positioning cameras, and starting data recording. Specific actions involved manipulating manual valves, activating the data acquisition system and pumps, and configuring gas injection durations through LabVIEW commands.

This meticulously arranged experimental setup enabled us to amass a comprehensive dataset, shedding light on the various aspects of gas-liquid two-phase flow regimes and offering significant insights into the dynamics of such flows in a controlled environment.

The two experimental scenarios explored were: static scenario (the Tower lab is filled with water to a bottom hole hydrostatic pressure of about 60 psig and not circulating) [7,8] and a dynamic scenario (water is circulating at a particular average flow rate typically 7 GPM or 15 GPM) [6,9]. Another controlled parameter during the experiment is the Kick Influx Pressure (KIP). Following safety considerations, the Tower lab is limited to an operating pressure of about 100 psig. Thus, the KIP ranged from 70 psig to 95 psig. The influx period ranged from 10 to 120 seconds based on expected flow regimes. As the influx period increases the gas void faction increases thus an evolution in the different gas-liquid two-phase flow regimes.

### 4.2 Python Script Development for Video Analysis

To enhance our analysis, we developed Python scripts tailored to examine the captured video footage frame-by-frame. These scripts employed advanced image processing techniques to isolate and extract high-resolution images of bubble migration within different two-phase flow regimes, including bubbly, slug, churn, and Taylor flows. By automating the detection and classification of flow patterns, the scripts enabled a more nuanced understanding of flow dynamics, facilitating the quantitative analysis of bubble size distribution, velocity, and phase interaction. This approach significantly augmented our dataset, providing a richer, more detailed perspective on the complex behaviors characteristic of gas-liquid two-phase flows.

The code retrieves the video file from the specified path, it then calculates the number of frames in the video file. The code has the flexibility to get a certain number of frames from the video as per the requirement. From a video the list of frames is extracted and stored in a list data structure. The code iterates through each frame and verifies if the frame was read correctly. Each frame is stored as a NumPy array. Each frame in the list is then converted into a .png format image using *imwrite* function of OpenCV library and stored in the folder as per specified path. The frames are named in the same convention as the videos, i.e. for air, psig_sec_floor_px, where psig, sec and floor are the details at which the video was shot and px stands for the picture x, x is the number of pictures.

## Limitations

This dataset, while extensive, has several limitations that we must acknowledge. Firstly, although each flow regime category contains over a hundred samples, a larger dataset would enhance the robustness and applicability of the data, particularly for machine learning applications where diversity and volume of data significantly impact model accuracy. Secondly, the absence of images capturing the "annular" flow regime is a notable gap; including such images would provide a more comprehensive view of two-phase flow dynamics, enriching the dataset further. Lastly, the exclusive focus on vertical flow loop conditions means the dataset lacks representation of horizontal or inclined two-phase flow regimes. This limitation restricts the dataset's utility for training models intended to recognize flow patterns in non-vertical orientations, potentially limiting its applicability across different engineering and research contexts.

## Ethics Statement

The authors have read the ethical requirements for publication in Data in Brief and confirm that the current work does not involve human subjects, animal experiments, or any data collected from social media platforms.

## Credit Author Statement

**Kaushik Manikonda:** Conceptualization, Methodology, Validation, Investigation, Resources, Writing - Original Draft, Supervision, Writing - Review & Editing, Project administration, **Chinemerem Obi:** Validation, Investigation, Resources, Writing - Original Draft, Writing - Review & Editing, Project administration, **Aarya Abhay Brahmane:** Software, Formal analysis, Data Curation, Writing - Original Draft, Writing - Review & Editing, **Mohammad Azizur Rahman:** Funding acquisition, Writing - Review & Editing, **Abu Rashid Hasan:** Funding acquisition, Writing - Original Draft, Writing - Review & Editing, Supervision, Project administration.

## Data Availability

Mendeley DataVertical Two-Phase Flow Regimes in an Annulus Image Dataset - Texas A&M University (Original data).

## References

[1] Hasan A.R., Kabir C.S. (2018).

[2] Manikonda K., Hasan A.R., Barooah A. (2020). *Proc.,* Abu Dhabi International Petroleum Exhibition & Conference.

[3] Manikonda K., Hasan A.R., Obi C.E. (2021). *Proc.,* Abu Dhabi International Petroleum Exhibition & Conference.

[4] Johnson A.B., White D.B. (1991). Gas-rise velocities during kicks. SPE Drilling Eng..

[5] Johnson A., Rezmer-Cooper I., Bailey T. (1995). *Proc.,* SPE/IADC Drilling Conference.

[6] Obi C.E., Hasan A.R., Abril L. (2022). Proc..

[7] Obi C.E., Hasan A.R., Luis A. (2022). *Proc.*, Thermal and Fluids Engineering Conference (TFEC), Partially Online Virtual and in Las Vegas, NV.

[8] Obi C.E., Yusuf F., Kaushik M. (2022). *Proc.,* SPE Western Regional Meeting.

[9] Obi C.E., Hasan A.R., Yusuf F. (2022). *Proc.,* Offshore Technology Conference.

